# Taxonomy-aware feature engineering for microbiome classification

**DOI:** 10.1186/s12859-018-2205-3

**Published:** 2018-06-15

**Authors:** Mai Oudah, Andreas Henschel

**Affiliations:** 1 Khalifa University of Science and Technology, Abu Dhabi, United Arab Emirates; 2 New York University Abu Dhabi, Abu Dhabi, United Arab Emirates

**Keywords:** Feature engineering, Supervised machine learning, Microbiome, Classification

## Abstract

**Background:**

What is a healthy microbiome? The pursuit of this and many related questions, especially in light of the recently recognized microbial component in a wide range of diseases has sparked a surge in metagenomic studies. They are often not simply attributable to a single pathogen but rather are the result of complex ecological processes. Relatedly, the increasing DNA sequencing depth and number of samples in metagenomic case-control studies enabled the applicability of powerful statistical methods, e.g. Machine Learning approaches. For the latter, the feature space is typically shaped by the relative abundances of operational taxonomic units, as determined by cost-effective phylogenetic marker gene profiles. While a substantial body of microbiome/microbiota research involves unsupervised and supervised Machine Learning, very little attention has been put on feature selection and engineering.

**Results:**

We here propose the first algorithm to exploit phylogenetic hierarchy (i.e. an all-encompassing taxonomy) in feature engineering for microbiota classification. The rationale is to exploit the often mono- or oligophyletic distribution of relevant (but hidden) traits by virtue of taxonomic abstraction. The algorithm is embedded in a comprehensive microbiota classification pipeline, which we applied to a diverse range of datasets, distinguishing healthy from diseased microbiota samples.

**Conclusion:**

We demonstrate substantial improvements over the state-of-the-art microbiota classification tools in terms of classification accuracy, regardless of the actual Machine Learning technique while using drastically reduced feature spaces. Moreover, generalized features bear great explanatory value: they provide a concise description of conditions and thus help to provide pathophysiological insights. Indeed, the automatically and reproducibly derived features are consistent with previously published domain expert analyses.

**Electronic supplementary material:**

The online version of this article (10.1186/s12859-018-2205-3) contains supplementary material, which is available to authorized users.

## Background

Traditional microbiology, strongly influenced by Robert Koch’s postulates, focuses on studies of bacteria (often pathogens) in isolation, an endeavor successful only for less than 1% of bacterial strains. While isolates and whole genome sequenc- ing projects continue to be valuable, metagenomic culture-independent approaches have provided a complementary view and have led to a more differentiated perception of bacteria as being unexpectedly diverse and predominantly commensal and beneficial. They allow comprehensive views of community composition and dynamics. Consequently, we can attempt to identify a healthy equilibrium of the micro- biota and how diversions from that equilibrium can be characterized. E.g., which combination and which abundance patterns of microorganisms ensure the correct functioning of digestion, are resilient to pathogens, train our immune system etc.? Likewise, environmental health is to a large extend attributable to the associated microbiota. They keep ecosystems intact by performing chemical processes such as material transformation. Also, the role microbiota play in biogeochemical cycles of life sustaining chemical elements such as carbon, oxygen, nitrogen can not be understated. Last not least, microbial community function are of commercial interest. when optimizing agricultural productivity and bioreactor stability in bioenergy applications and biochemical engineering. In all these scenarios it is desirable to understand the taxonomic composition and function of those microbiota and the dynamics that influence their function. Recent advances in Next Generation DNA Sequencing have turned microbiome research into a very data-intensive field [1] thanks to steeply dropping costs of DNA sequencing and advances in multiplexing of many samples in metagenomic marker gene sequencing. In particular, compositional exploration is commonly carried out through tag sequencing, e.g. using hypervariable regions of the 16S rRNA gene.

It is remarkable that microbiota of the lower gut have been shown to be indicative for colorectal cancer [2, 3]. The abundance patterns of microbes as measured by tag-sequencing of taxonomic marker genes (16S rRNA profiles) facilitate the categorization of microbiota with respect to their function. Sophisticated classifiers are not required for those cases easily distinguishable through high prevalence of a pathogen (e.g. Clostridium difficile) or dysbiosis (drastic loss of alpha-diversity). Those cases can be addressed using simple statistical measures or unsupervised learning, for example. However, a large range of medical conditions that stretch far beyond infectious diseases are related to subtle compositional changes in microbial communities. A current ongoing debate is to what extend it is possible to robustly cluster microbiota into community types or enterotypes (with respect to the human gut [4]). The hope tied to the identification of those community−/enterotypes is that crisp, distinct clusters can be associated with a special functionality and thus lead to a better understanding of a microbiome related condition and in turn, more targeted therapeutics. Large scale studies like the Human Microbiome Project have shown that human microbiota cluster well by body site [5]. A similar observation was reported based on a meta-analysis for environmental microbiota clustering according to their ecosystem [6]. Those clusters are observable through dimensionality reduction methods (e.g. ordination methods such as Principal Coordinate Analysis) and unsupervised learning (e.g. hierarchical clustering).

However, microbiota associated to medical conditions like Colorectal Cancer (CRC), Inflammatory Bowel Disease (IBD), Crohn’s disease and (pre-)diabetes are not simply falling into clusters and classification of healthy and diseased microbiota samples is beyond unsupervised learning. On the other hand, Supervised learning, in particular Random Forests, Support Vector Machines and Boosting, have been applied successfully to a large set of microbiota classification problems [2, 7–10], but little attention has been devoted to feature selection and feature engineering. The common approach to design the feature space for Supervised Learning is the grouping of 16S rRNA sequence reads by Operational Taxonomic Unit (OTU) in order to reduce the dimensionality of the dataset from millions of sequences to thousands of OTUs. The relative OTU abundances then form the feature vectors representing the microbiota. Gut microbiota as well as other microbial communities in soil, marine environments etc. are rather complex in terms of alpha-diversity as we frequently observe thousands of OTUs in a single sample. This generally poses a very feature rich learning task. Further microbiota feature reduction could simply be achieved by lowering OTU resolution below the common 97% sequence identity (yielding fewer but more diverse taxonomic bins) or low abundance filtering by disregarding OTUs which are either appearing only in few samples or which are on average below a certain threshold. However, these crude measures are likely to cause loss of important information and are henceforth not considered viable for microbiota representations.

Despite the above mentioned increase of samples for a particular classification task, the high ratio of feature space dimensionality over dataset size still incurs the curse of dimensionality and with it the risk of overfitting. Classification with fewer but better features is therefore desirable, a concept commonly referred to as Feature Space Compression. Owing to the nature of NP-completeness, feature subset selection requires heuristic solutions for large feature spaces. Feature selection can be done by filter methods, wrapper methods or embedded methods [9]. Recent work on microbiota/metagenome classification, such as Fizzy [11] and MetAML [12], utilize standard feature selection algorithms, not capitalizing on the evolutionary relationship and thus the hierarchical structure of features. Fizzy implements a number of standard Information-theoretic subset selection methods (e.g. JMI, MIM and mRMR from FEAST C library), NPFS and Lasso. MetAML performs microbiota or full metagenomic classification, which incorporates embedded feature selection methods, including Lasso and ENet, with Random Forests (RF) and Support Vector Machines (SVM) classifiers.

In this study, we aim to distill informative features from datasets independently of the Machine Learning approach. In contrast to filter methods for feature selection, wrapper and embedded methods are often computationally expensive due to the reiteration of the training process. The state-of-the-art feature selection methods, in many cases, cannot handle the potential search space for the best subset of features in microbiota datasets. For example, the Correlation-based Feature Selection (CFS) [13], central part of the popular WEKA toolkit, does not scale well to the feature space dimension typically found in microbiota classification tasks with several thousand OTUs.

Kostic et al. [14] have described their findings with regard to microbiota in colorectal cancer in terms of genera and phyla, which showed that not only are bacterial taxa powerful predictors for important conditions, but they also lend themselves naturally to generalization due to their taxonomy. Remarkably, those high-level features pose a compact and human understandable biomarker formulation of a condition. Like in these examples, experts often discriminate between microbiata classes in terms of few taxa of various phylogenetic ranks based on manual inspection of few samples. However, it is often unclear whether the chosen terms are of the right level of generality, i.e. of the most suitable rank.

The goal of this work is to formalize this process by systematically creating and reproducibly searching a suitable hypothesis space. In this context it is important to note that features in microbiota classification and in Machine Learning tasks in general are often not independent. To exploit this, we can borrow from advances in the Knowledge Management community, where general-to-specific ordered concept taxonomies are used to describe nouns, common features of text documents. Ristoski and Paulheim have recently presented an algorithm that performs feature selection given an underlying hierarchy for the features. The authors have shown that their algorithm outperforms other hierarchy based and non-hierarchy based feature selection methods [15].

However, the existing hierarchical feature selection algorithms, as in by Ristoski et al. [15], only deal with binary features (i.e. presence-absence representations), which fail to adequately represent biological data in high resolution and cause a high loss of information compared to relative abundances. Moreover, partial 16S rRNA sequences are often assigned taxonomic ranks to genus or family level with sufficient certainty, which makes their hierarchical information often incomplete. In this paper, we introduce a hierarchical feature engineering (HFE) method, which goes beyond mere feature selection. HFE exploits the underlying hierarchical structure of the feature space in order to create an extended version of the feature space to start with, which will go through a number of processing steps resulting in a much smaller space of informative features for supervised machine learning.

In summary, while hierarchical feature engineering seems a promising approach to Feature Space Compression, adjustments for the type of data in microbiota classification tasks are required.

## Methods

**Fig. 1:**
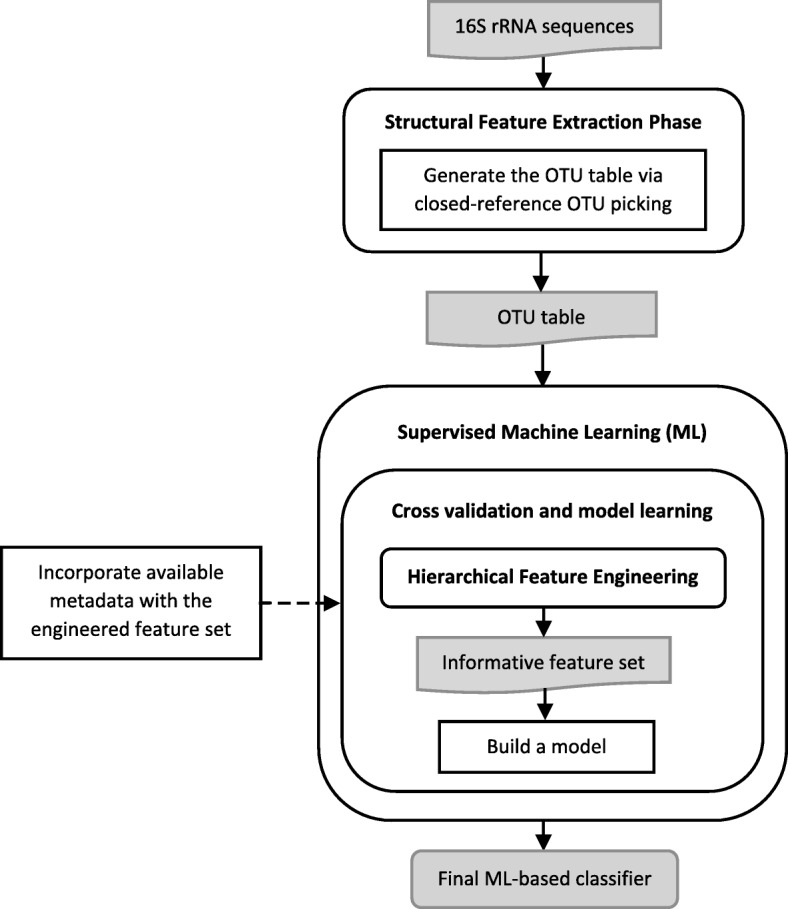
Proposed pipeline for metagenome classification

The introduced pipeline for Microbiota classification is composed of three main phases, including 1) Structural Feature Extraction, 2) Hierarchical Feature Engineering, and 3) Supervised Machine Learning, as illustrated in Fig. 1.

### Structural feature extraction

The structural features, which represent the bacterial composition of a microbial community, comprise the main feature space for microbiota samples. Those features are derived from the 16S rRNA sequences via closed-reference Operational Taxonomic Unit (OTU) picking procedure provided by QIIME [16], an open-source tool for microbiome analysis. In closed-reference OTU picking, only sequences with hits in the reference sequence database of GreenGenes are used to construct the OTU table, which consists of a list of OTUs and their abundances per sample. We chose closed-reference OTU picking because it allows to combine datasets with different variable regions. The taxonomy of the identified microbiome is automatically constructed from the predefined taxonomy of the OTU representatives in the reference sequence database. A taxonomy lineage of an OTU is composed of 7 taxonomic ranks: Kingdom, Phylum, Class, Order, Family, Genus and Species, respectively from the highest to the lowest level. We add an eighth level to the bottom of the hierarchy to represent the OTU level.

### Hierarchical feature engineering

**Fig. 2:**
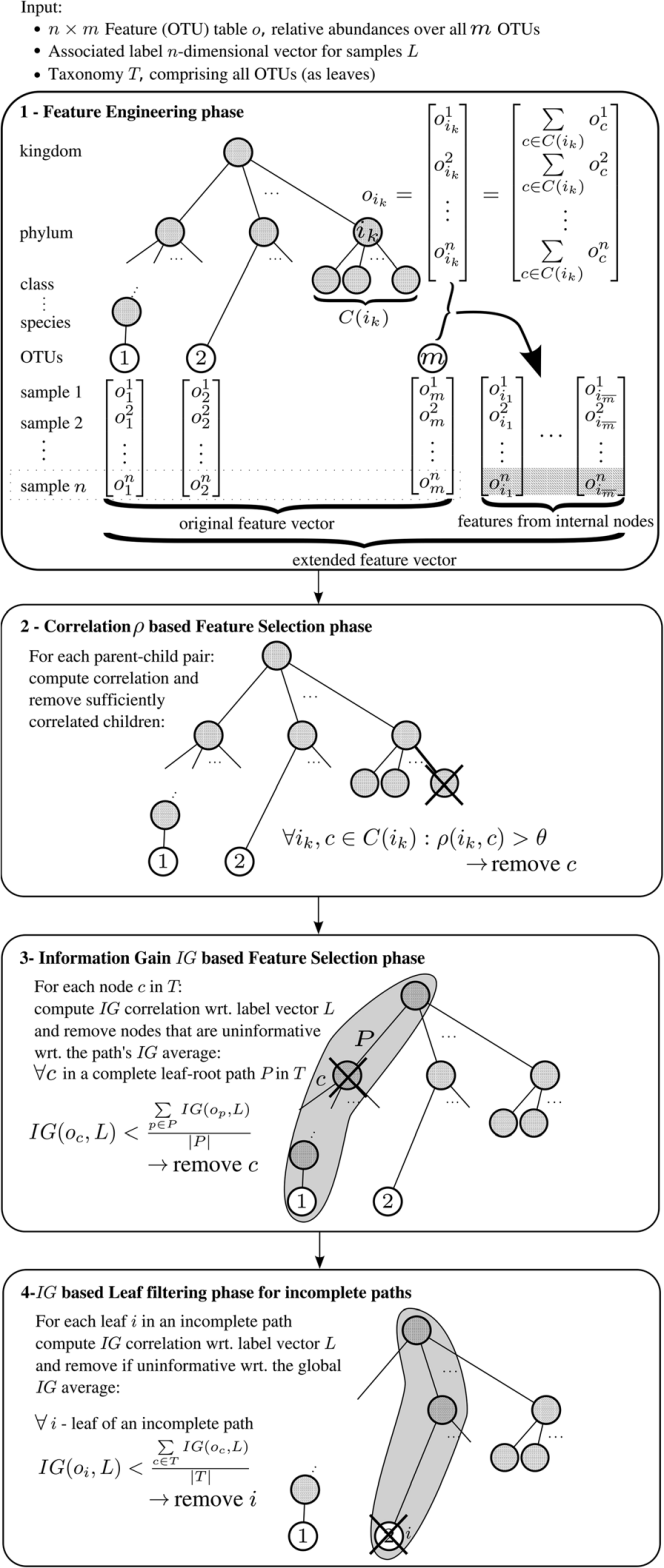
The HFE algorithm. Note that OTUs are possibly associated to higher taxonomic ranks (e.g. OTU 2) due to incomplete taxonomic classification. We refer to them as leaves in incomplete paths. The feature space first grows from R^*m*^ to R^*m*^^ + ^^*m'*^, where *m'* is the number of internal nodes in *T* (phase 1). Subsequently the feature space is reduced by the number of sufficiently correlated child nodes (*s*_1_, phase 2) and relatively uninformative features (*s*_2_ and *s*_3_, phase 3 and 4, resp.), yielding the final feature space R^*m*^^ + ^^*m − s*^^1^ ^*− s*^^2^ ^*− s*^^3^. The *n* samples represent the training dataset

The basic architecture of the HFE method is inspired by Ristoski et al.'s [15] work on feature selection in hierarchical feature space. The input to HFE is composed of three items: 1) The -transposed- OTU table *o*, where rows represent the *n* samples from the training dataset allocated for building a ML model (i.e. the samples available within 9 partitions out of 10 in 10-fold cross validation, while the 10^th^ partition is put aside for testing that ML model. The process is repeated 10 times, so that each partition is to serve as a testing dataset one time), and columns represent the *m* features, (i.e. the OTUs from the OTU table); 2) the associated *n*-dimensional label vector indicating the predefined class of each sample from the training dataset, e.g. cancer or normal; and 3) the taxonomy *T*. Our HFE method consists of four phases, as shown in Fig. 2, including:

1. **Feature engineering phase:** We consider the relative abundances of higher taxonomic units *ik* as potential features by summing up the relative abundances of their respective children *C* in a bottom up tree traversal: *o*_*ik*_ =Σ_*c*__∈__*C*__(__*ik*__)_ *o*_*c*_.

2. **Correlation-based filtering phase:** For each parent-child pair in the hierarchy, the Pearson correlation coefficient *ρ* is calculated from the parent and child vectors of values over all samples. If the result is greater than a predefined threshold *θ*, then the child node is discarded. Otherwise, the child node is kept as part of the hierarchy. It is worth mentioning that we aim to remove child nodes that are redundant to their parents, for which Pearson correlation serves as a proxy. Friedman et al. [17] states that the compositional effect for detecting spurious correlations is less for complex communities (with thousands of OTUs), which is what our method is directed at. Moreover, we use correlation simply as a heuristic to select features, as opposed to microbial network reconstruction.

3. **Information Gain (*IG*) based Filtering Phase:** Based on the retained nodes from the previous phase, all paths are constructed from the leaves to the root (i.e., each OTU’s lineage). For each path, the *IG* [18] of each node on the path is calculated with respect to the labels/classes *L*. Then the average *IG* is calculated and used as a threshold to discard any node with lower *IG* score or an *IG* score of zero. Note that this does not apply to leaves of incomplete paths, which are dealt with in phase 4. As the *IG* measure is originally designed to handle discrete (categorical) features and ours are continuous features, a step of discretization is applied via WEKA on the features prior to *IG* computing. Note that WEKA’s information gain calculation for continuous features is based on supervised multi-interval discretization, as described in Fayyad et al. [19]. This way, our classification algorithm can handle not only continuous features, but also multiple classes.

4. *IG*-**based Leaf Filtering Phase:** In order to handle OTUs with incomplete taxonomic information, i.e. those OTUs for which taxonomic classification could not be completed with high confidence all the way down to species level, we introduce a fourth phase dealing with incomplete paths, which dis- cards any leaf with an *IG* score less than the global average *IG* score of the remaining nodes from the third phase or an *IG* score of zero. There is no constraint on the percentage of discarded features in this phase. Many tax- onomically underspecified OTUs would be retained without this additional filter, as they do not correlate with remote ancestors and often have higher information gain then the average of the few high level taxa in their lineage. The empirical results show that adding the features selected by the fourth phase to the output of the third phase has improved the overall performance of the produced classification model when used for CRC detection.

The resultant is a set of informative features, including OTUs and elements of the taxonomy, which can be utilized for supervised ML. Furthermore, metadata can be added to the final feature set. The introduced HFE method, which is implemented in Python, differs from the method of Ristoski et al. [15] in two main aspects:

1. **The targeted type of features:** Ristoski et al. [15] designed a method for feature spaces of binary attributes (presence/absence), while our HFE method can handle feature spaces of continuous attributes, such as relative abundances.
2. **The ability to handle incomplete or missing hierarchical information:** The method by Ristoski et al. [15] is designed to handle attributes with complete hierarchical information, while our HFE method introduces the phase 4, i.e. *IG*-based leaf filtering phase, which handles attributes with missing ranks in the hierarchy.

### Supervised machine learning

The final phase in the proposed pipeline is learning and evaluating a classification model via a ML algorithm utilizing the HFE and 10-fold cross validation, where the HFE method is applied separately for each cross validation fold. In this component, any ML algorithm for classification is applicable. We use WEKA [20], a comprehensive workbench with support for a large number of ML algorithms, as the development environment for the classification models. A sample from an example input to the ML-based component is illustrated in Table 1, where the columns represent the features and the rows represent the microbial community samples.

**Table 1:** Sample from a final feature set. Note that it contains original features (OTUs with numerical identifiers) and high level taxa

s_obeum	1075307	192066	g_Coprococcus	g_Streptococcus	s_bromii	360660	class
0.00	186.34	0.00	1925.47	186.34	0.00	124.22	Cancer
169.49	24.21	72.64	1573.85	145.28	605.33	72.64	Normal
105.26	105.26	0.00	526.32	1684.21	842.11	0.00	Normal
8.64	0.00	0.27	1578.41	2869.76	327.29	0.27	Cancer
0.00	299.63	299.63	786.52	636.70	0.00	0.00	Cancer
107.66	0.00	0.00	1148.33	2571.77	0.00	83.73	Normal
8.48	0.00	0.53	2575.43	341.47	715.25	0.00	Cancer
29.33	29.33	0.00	1261.00	87.98	821.11	29.33	Normal

The numbers represent relative abundances multiplied by 10^5^

## Results

In this section, we evaluate the performance of the proposed methodology when applied on real biological datasets from different studies. Moreover, we conduct a comparison between our HFE method and other tools incorporating feature selection methods on biological datasets.

### Experimental settings

For each dataset, the initial feature set is the OTU table generated via the Structural Feature Extraction Phase of the introduced pipeline. It is noteworthy that we use the same version of GreenGenes with all datasets, i.e. May 2013 GreenGenes (GG version 13.5). The performance of the classification model trained on the initial feature set of a dataset is considered the baseline for comparison.

For each initial feature set, conventional unsupervised learning, represented here by Principle Coordinate Analysis (PCoA) technique, is utilized to cluster similar samples together in order to distinguish between the different groups of samples. Studies, where the unsupervised learning is sufficient for clear group separation, are exploited for validation. Otherwise,the studies are used to demonstrate and evaluate the capabilities of the introduced pipeline with more complicated classification tasks. The PCoA calculations and plots are produced by QIIME (version 1.8.0). Three different values, i.e. 0.6, 0.7 and 0.8, that constitute for a strong correlation have been examined as correlation threshold *θ*, a free parameter in HFE, when applied on initial datasets in order to generate reduced sets of informative features. The classification results show no significant difference in performance among the three values when utilized by the Correlation-based Filtering Phase in HFE. Therefore, the default value for *θ* is set to 0.7. The classification models are generated under WEKA environment, where the default settings of the selected ML algorithms are used, via 10-fold cross validation [21] in order to avoid overfitting. The results are presented in terms of the area under the Receiver operating characteristic (ROC) curve, i.e. AUC, precision (P), Recall (R) and F-measure (F). The significance of the differences in performance between the proposed method (HFE) and other strategies is illustrated through the p-value calculated via conducting a statistical t-test, in which a p-value less than 0.05 constitutes a significant improvement in the performance.

#### Machine learning algorithms

A number of variant ML algorithms are examined as part of the Supervised ML component, including Decision Trees (DT), Random Forests (RF) and Naïve Bayes (NB) algorithms [20, 21], to demonstrate the improvement in the accuracy achieved regardless of the ML algorithm. WEKA has implementations of the selected ML algorithms, including J48, RandomForest and NaiveBayes built-in classifiers, respectively. We use the python-weka-wrapper (version 0.3.6) library, which enables the use of WEKA from within Python.

### 16S rRNA sequence datasets

The biological datasets utilized for the pipeline performance’s evaluation are NGS based 16S rRNA sequence profiles provided by metagenomics studies, using universal primers and suitable for classification.

#### Human body site prediction

For the classification task of Human Body Site prediction, we use the initial dataset HMPv35 100nt even1k, i.e. an OTU table via closed-reference OTU picking against GG 13.5 with the sequences being trimmed to 100 nucleotides prior to OTU picking and then rarified to 1000 sequences per sample, provided by the Human Microbiome Project [22]. The dataset is composed of 4,845 samples taken from 5 human body sites: Airways, Skin, Oral, Gastrointestinal tract and Urogenital tract. The initial feature set consists of 5,430 OTUs.

#### Environment prediction

For the classification task of Environment Prediction, we use the initial dataset, i.e. an OTU table via closed-reference OTU picking against GG 13.5, provided from the Meta-analysis of environmental microbiomes done by Henschel, Anwar and Manohar [6]. The dataset is composed of 10,101 samples categorized into 24 (singular and composite) environments. The main environments are Soil, Marine, Freshwater, Biofilm, Plant associated, Animal/Human associated, Anthropogenic, Geothermal and Hypersaline. The initial feature set consists of 30,860 OTUs.

#### Colorectal Cancer detection

Colorectal cancer (CRC) is the third most common type of cancer around the world, and it is responsible for losing over half a million people every year [23]. Developing effective screening methods can be crucial for early detection and increasing the survival rate. Nowadays, fecal occult blood test (FOBT) is commonly used as the screening technique for CRC [2, 3], but due to its limited accuracy, there is still a need for a more reliable noninvasive screening method. In this study, we apply our pipeline on two CRC datasets that have been built to explore the potential of using the microbiome from fecal samples for CRC screening:

- The first CRC dataset (CRC1) is available from Zeller et al.'s [2] study. It is composed of 90 cancer samples and 92 control samples. The initial feature set consists of 18,170 OTUs.
- The second CRC dataset (CRC2) is available from Zackular et al.'s [3] study. It is composed of 30 cancer samples and 30 control samples. The initial feature set consists of 6807 OTUs.
  Moreover, we have built a combined CRC dataset (CRC1+2) of the above two CRC datasets in order to build a larger dataset in terms of number of samples, due to being a desired dataset property for ML. The initial feature set consists of 19,009 OTUs.

### Empirical results

The Human Microbiome Project Consortium's [22] dataset (for characterizing the microbiota of various human body sites) and Henschel et al.'s [6] dataset (for characterizing the microbiota of various environments) can be handled by unsupervised machine learning, as shown in Additional file 1: Figures S1 and S2, to distinguish between samples of different groups with acceptable performance. Albeit an easy learning task, we use HFE in order to show its applicability to diverse, large scale datasets. Additional file 1: Table S1 illustrates the pipeline’s cross validation results when applied to the two datasets in terms of AUC. DT, RF and NB are utilized as the selected ML algorithms in the supervised ML component. HFE compares well to the baseline but, importantly, uses substantially less features.

**Fig. 3:**
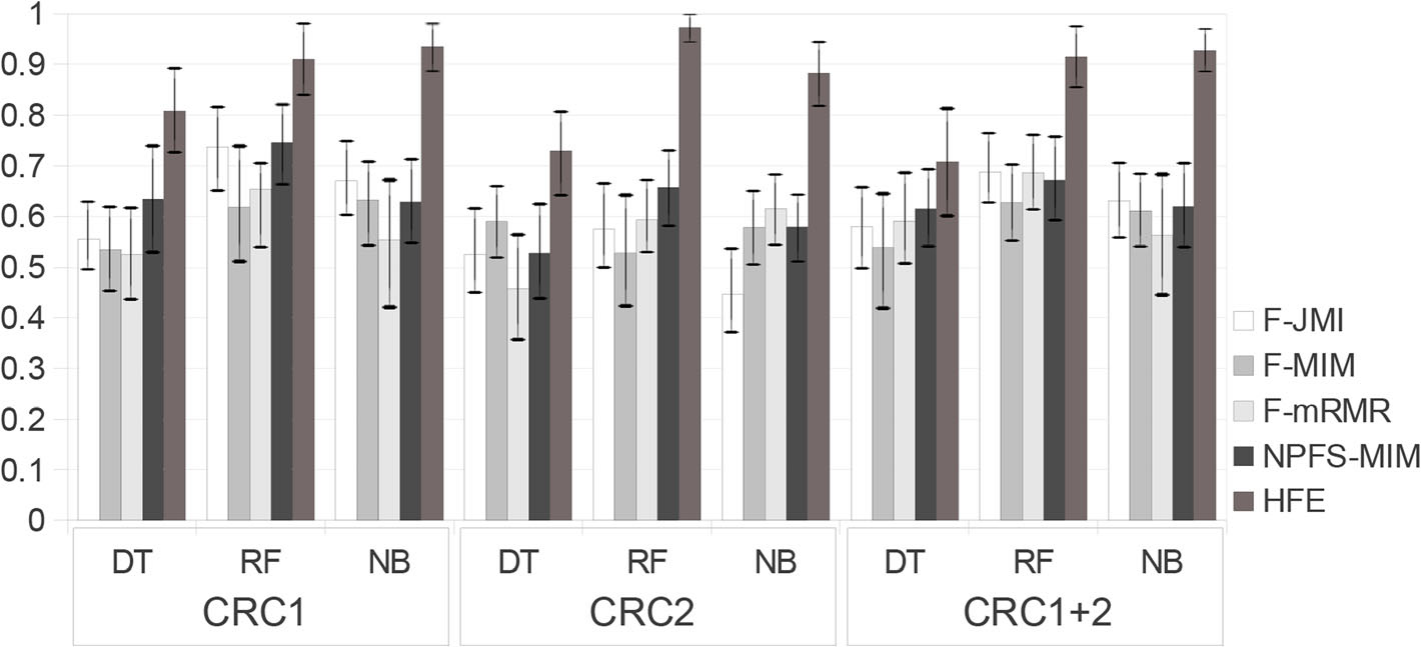
AUC comparison of HFE vs. Fizzy when applied to the CRC datasets

In the case of CRC detection, unsupervised learning cannot clearly distinguish between samples of different groups, as illustrated in Additional file 1: Figure S3. Tables 2, 3 and 4 show the pipeline’s classification results when applied on the CRC datasets (CRC1, CRC2, CRC1+2, respectively), in terms of AUC, precision, recall and f-measure, compared to the baseline results when no feature selection was used. The variability among the different folds of cross validation is captured via the standard deviation of AUC, precision, recall, f-measure and the size of the engineered feature sets, which shows standard deviation scores that range from 0.074 to 0.217 for the evaluation scores, and a range from 6.633 to 12.328 when it comes to the size of the feature subsets across folds in the CRC studies. In the largest CRC dataset, i.e. CRC1+2, in particular, the mean and standard deviation of the feature subset size across folds are 96 and 6.633 (variance *≈* 44), respectively, while the size of the feature subset intersection obtained across folds is 31 features. Comparing variability results among the CRC datasets shows that the larger the feature space the smaller the variance of the feature subset size and the larger the feature subset intersection obtained across folds. For building the classification models for both baseline and HFE feature sets, we consider DT, RF and NB algorithms for their ability to computationally handle a varied range of feature space under WEKA framework. The results in Table 2 through Table 4 show that using HFE improves the performance in general across the CRC datasets when compared to the baseline performance, especially using Random Forest as the ML algorithm. It is worth mentioning that we have conducted a comparison between the performance of the proposed method with and without the 4^th^ phase, i.e. responsible for handling features with incomplete hierarchical information, in order to examine whether it adds value to the pipeline, and the results showed that the features selected by the 4^th^ phase improves the quality of the performance in terms of AUC, by 3.7%, 12% and 6.7% when applied to CRC1, CRC2 and CRC1+2, respectively, using RF as the ML algorithm. Moreover, we conduct a comparison among Fizzy, MetAML and HFE, in terms of AUC and number of selected features, of which DT, NB and RF are used for building the classification models. Fig. 3 and Table 5 illustrate the comparison between Fizzy and HFE when applied to the CRC datasets using several ML algorithms, which shows significant improvements in the performance using HFE over Fizzy’s feature selection algorithms (JMI, MIM, mRMR and NPFS-MIM), with p-value of 0.0007, 0.0035 and 0.0358 for Fizzy-(JMI/MIM/mRMR) vs. HFE when applied on CRC1, CRC2 and CRC1+2, respectively, and an overall p-value of 0.0494 for NPFS-MIM vs. HFE when applied to the CRC datasets. It should be noted that Fizzy requires the number of features to be predefined ahead to the actual feature selection process, except for NPFS. Therefore we assign sizes that are similar to the ones produced by HFE, i.e. 97, 28 and 92 for CRC1, CRC2 and CRC1+2, respectively, and then ones that are slightly above those of HFE, i.e. 110, 50 and 100, respectively as well, to allow for comparison. The number of features selected by NPFS-MIM for each CRC dataset is 654, 167 and 513, respectively. Moreover, Table 6 compares the performance of MetAML vs. HFE when applied to the CRC and IBD datasets provided by Pasolli et al. [12], which are taxonomic profiles at species-level generated from shotgun sequencing data. The results show that HFE outperforms the best results achieved by MetAML in terms of AUC, with a p-value of 0.0492, when RF is used with/without embedded feature selection methods, i.e enet and lasso, while using far less features. Note that HFE also overcomes MetAML’s limitation to deal with complete taxonomic information. We thus expect the performance margin to increase further, when including OTUs with incomplete taxonomic lineages to the dataset. Additional file 1 Figure S4 through Additional file 1: Figure S7 illustrate comparisons between the confusion matrices of the CRC datasets’ baseline models and HFE-based models with respect to the same set of algorithms, and show how the performances are improved with the use of HFE regardless of the number of categories in the classification task, i.e. Cancer vs. Normal or Cancer vs. Normal vs. Adenoma.

**Table 2:** The performance of the proposed pipeline when applied on CRC1 dataset, in terms of mean AUC, precision (P), recall (R) and F-measure (F), and their standard deviation

		BL				HFE			
		AUC	P	R	F	AUC	P	R	F
DT	Score	0.635	0.621	0.621	0.620	0.678	0.707	0.676	0.675
	std.	0.130	0.150	0.170	0.130	0.103	0.105	0.105	0.096
NB	Score	0.686	0.678	0.676	0.674	0.721	0.715	0.704	0.705
	std.	0.110	0.130	0.150	0.110	0.116	0.113	0.102	0.103
RF	Score	0.770	0.677	0.676	0.675	0.795	0.728	0.709	0.706
	std.	0.110	0.130	0.150	0.100	0.143	0.125	0.107	0.111
Features		count: 18,170				count: 97 std.:12.328

**Table 3:** The performance of the proposed pipeline when applied on CRC2 dataset, in terms of mean AUC, precision (P), recall (R) and F-measure (F), and their standard deviation

		BL				HFE			
		AUC	P	R	F	AUC	P	R	F
DT	Score	0.697	0.700	0.700	0.700	0.657	0.692	0.617	0.582
	std.	0.160	0.210	0.270	0.200	0.158	0.226	0.150	0.174
NB	Score	0.647	0.650	0.650	0.650	0.583	0.566	0.550	0.515
	std.	0.200	0.290	0.290	0.250	0.217	0.214	0.150	0.163
RF	Score	0.669	0.674	0.667	0.663	0.975	0.915	0.883	0.884
	std.	0.200	0.240	0.290	0.220	0.075	0.104	0.130	0.130
Features		count: 6807				count: 28 std. 10.677

**Table 4:** The performance of the proposed pipeline when applied on CRC1 + 2 dataset, in terms of mean AUC, precision (P), recall (R) and F-measure (F), and their standard deviation

		BL				HFE			
		AUC	P	R	F	AUC	P	R	F
DT	Score	0.630	0.625	0.625	0.625	0.588	0.600	0.561	0.566
	std.	0.110	0.100	0.140	0.100	0.108	0.116	0.115	0.115
NB	Score	0.647	0.655	0.657	0.654	0.731	0.701	0.654	0.657
	std.	0.100	0.090	0.140	0.090	0.164	0.132	0.113	0.113
RF	Score	0.736	0.670	0.667	0.654	0.809	0.753	0.737	0.738
	std.	0.110	0.080	0.110	0.080	0.074	0.090	0.091	0.092
Features		count: 19,009				count: 92 mean: 96 std.: 6.633

**Table 5:** Comparison between the performance of our HFE method and Fizzy when applied to the CRC datasets, in terms of mean AUC

		CRC1 #Features		CRC2 #Features		CRC1 + 2 #Features	
	ML	110	97	50	28	100	92
F-JMI	DT	0.555	0.561	0.526	0.594	0.580	0.612
	NB	0.670	0.672	0.446	0.350	0.631	0.611
	RF	0.737	0.749	0.574	0.418	0.688	0.632
F-MIM	DT	0.535	0.548	0.590	0.557	0.539	0.561
	NB	0.632	0.588	0.578	0.422	0.611	0.637
	RF	0.618	0.563	0.528	0.374	0.627	0.675
F-mRMR	DT	0.525	0.530	0.458	0.557	0.591	0.623
	NB	0.553	0.544	0.615	0.614	0.562	0.559
	RF	0.653	0.665	0.593	0.540	0.686	0.705
#Features by NPFS		654		167		513	
NPFS-MIM	DT	0.634		0.528		0.615	
	NB	0.628		0.579		0.619	
	RF	0.746		0.657		0.671	
#Features by HFE		97		28		92	
HFE	DT	0.680		0.660		0.590	
	NB	0.750		0.583		0.731	
	RF	0.800		0.980		0.810

**Table 6:** Thw performance of HFE vs. MetAML when applied to the CRC and IBD datasets provided by Pasolli et al. [12], in terms of mean AUC and standard deviation

	CRC_MetAML_			IBD_MetAML_		
	RF			RF		
	Mean	Std.	#Features	Mean	Std.	#Features
MetAML-RF	0.874	0.102	490	0.891	0.100	434
MetAML-RF-Enet:Emb	0.870	0.108	70	0.898	0.103	125
MetAML-RF-Lasso:Emb	0.769	0.135	90	0.883	0.118	70
HFE	0.910	0.093	34	0.919	0.090	26

**Fig. 4:**
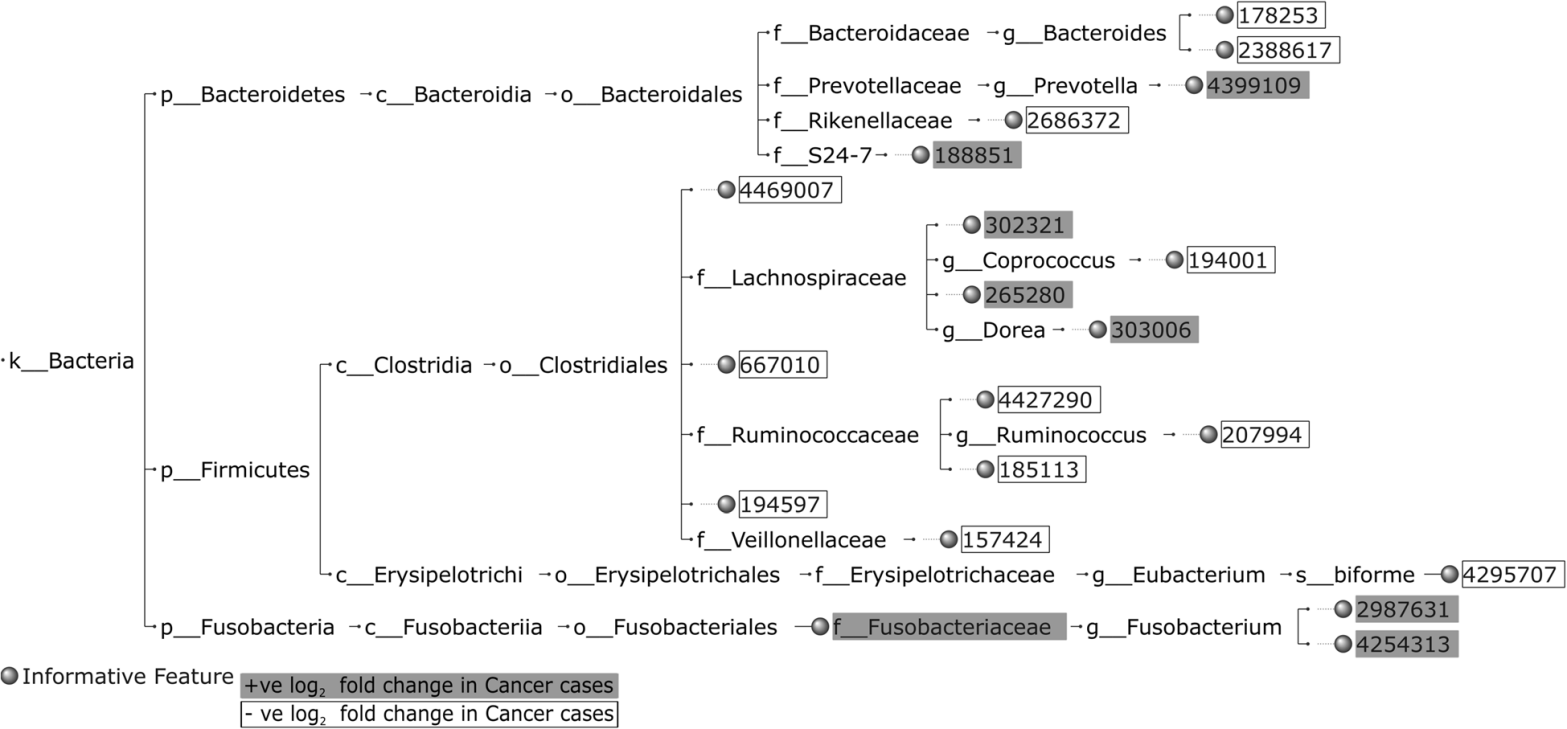
The taxonomic tree of the top 20 informative features extracted by the HFE method, in terms of IG, for Cancer vs. Normal classification for CRC1

**Fig. 5:**
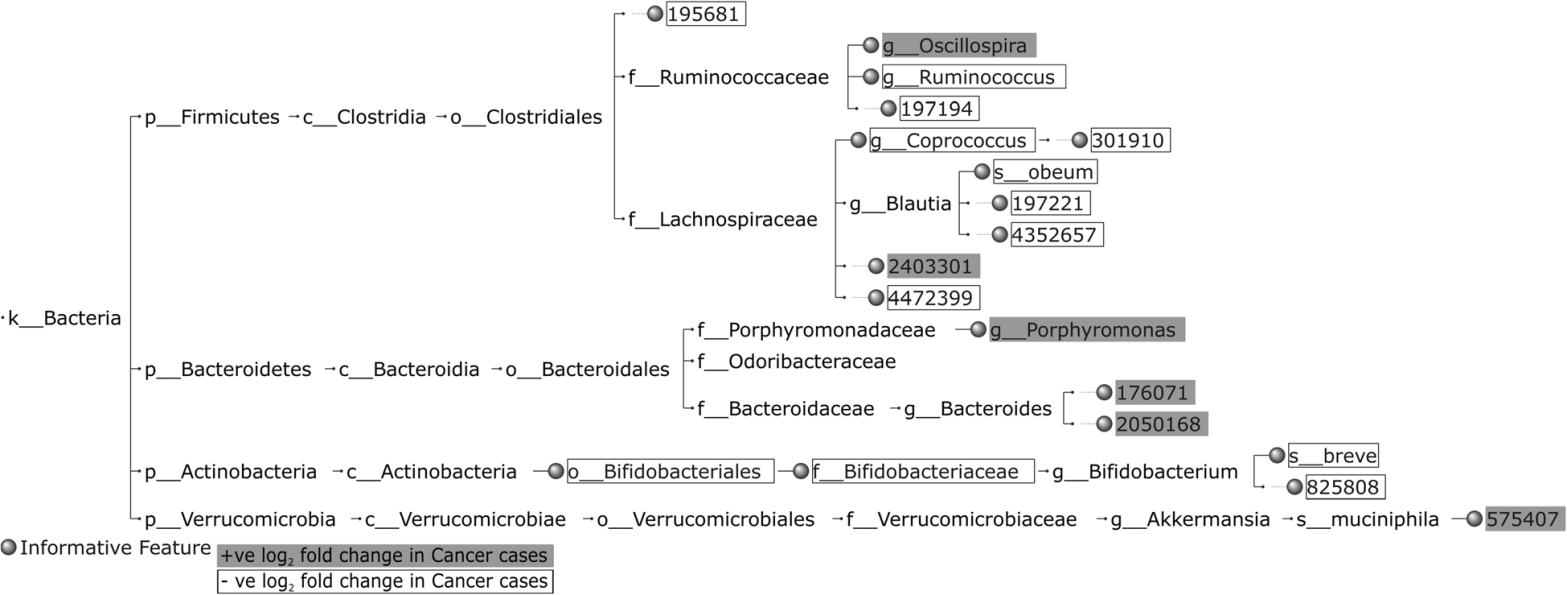
The taxonomic tree of the top 20 informative features extracted by the HFE method, in terms of IG, for Cancer vs. Normal classification for CRC2

**Fig. 6:**
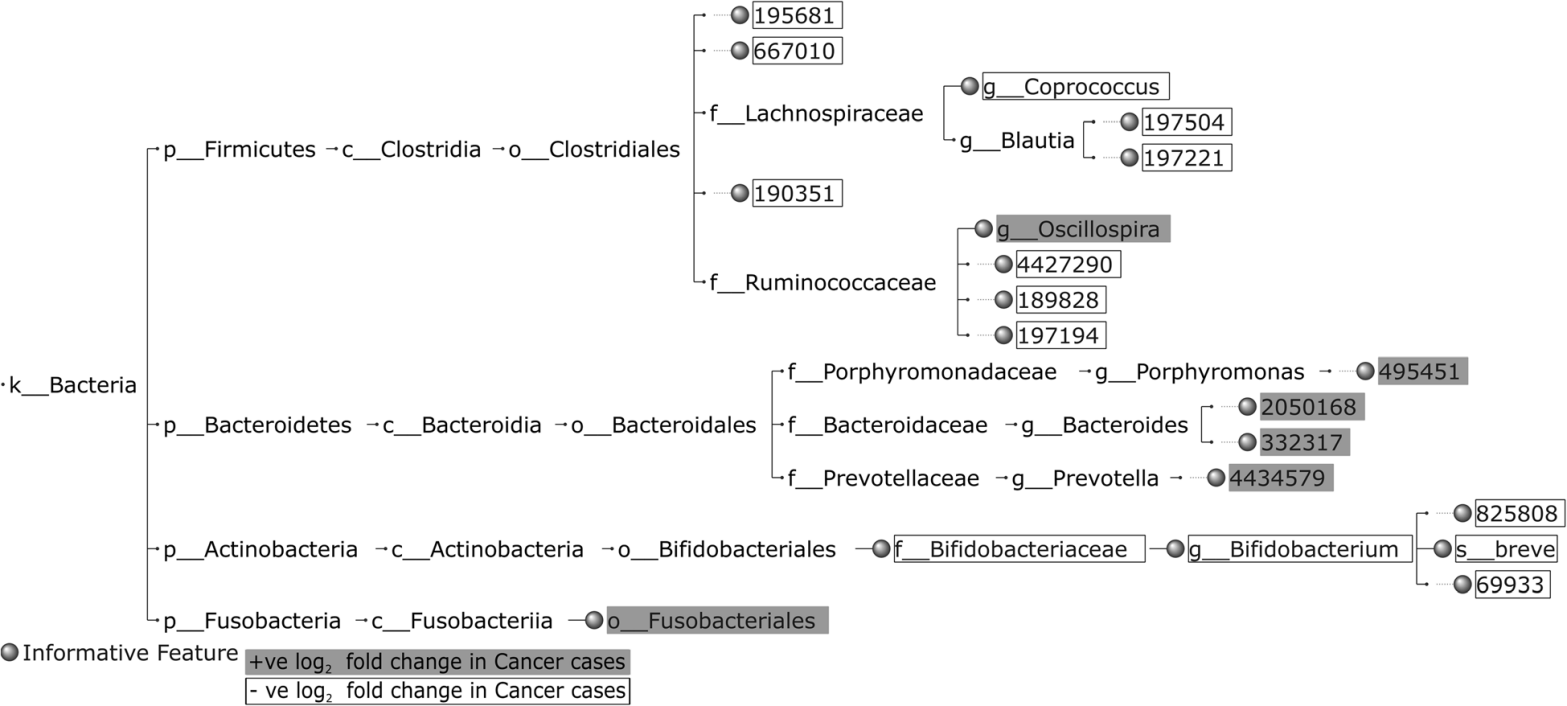
The taxonomic tree of the top 20 informative features extracted by the HFE method, in terms of IG, for Cancer vs. Normal classification for CRC1 + 2

Figures 4, 5 and 6 highlight the top 20 HFE informative features for Cancer vs. Normal classification, in terms of IG score, and the nature of their *log*_2_ fold change (positive (+ve)/negative (-ve)) across the CRC datasets. Some of the common HFE informative features between CRC1 and CRC2 datasets can be found in the top 20, including OTUs and/or taxonomic units associated with *Coprococcus*, *Ruminococcaceae*, *Bacteroides*, *Lachnospiraceae* and *Clostridiales*, which are reflected in the informative feature set of the combined CRC dataset (CRC1+2) as well, supporting the attempt to constitute a generalized feature set for CRC detection. Moreover, our findings per dataset are comparable with those of the original studies. For CRC1, both our and Zeller et al.'s [2] results with regard to informative features include OTUs asso- ciated with *Fusobacteriaceae*, *Peptostreptococcus*, *Clostridium*, *Bacteroides*, *Lactobacillus*, *Eubacterium*, *Bifidobacterium*, *Dorea*, *Lachnospiraceae*, *Ruminococcus* and *Streptococcus*. For CRC2, both our and Zackular et al.'s [3] results with regard to informative features include OTUs associated with *Fusobacterium*, *Bacteroidales*, *Lachnospiraceae*, *Gammaproteobacteria*, *Bacteroides*, and *Clostridiales*. It is worth noting that the informative feature set of the combined CRC dataset encloses a number of OTUs, which have not been reported specifically by Zeller et al. and Zackular et al. [2, 3], including *Fusobacteriales*, *Oscillospira* and some OTUs associated with *Porphyromonas*, *Rikenellaceae*, *Prevotella*, *Akkermansia muciniphila*, *Lawsonia*, and *S24-7* of Bacteroidales, which are highly presented in Cancer samples, and *Yaniellaceae*, Cellulomonadaceae, *Coprococcus*, *Bifidobacteriaceae*, *Bifidobacterium breve*, *Bacilli*, *Lactobacillus ruminis*, *Lactobacillus delbrueckii*, *Rhizobiales*, and some OTUs associated with *Blautia* and *Blautia producta*, which are highly presented in Normal samples. The observation of OTUs assigned to *Akkermansia muciniphila* to be overrepresented in Cancer samples is particularly intruiging as *Akkermansia muciniphila* has been associated with healthy metabolism [24, 25].

## Discussion

It seems plausible that taxa on all ranks potentially make for good features in microbiota classification: each rank subsumes different spectra of organisms that share traits that are encoded by predominantly vertically inherited genes from a shared ancestor. The proposed Hierarchical Feature Engineering (HFE) method exploits the intrinsic hierarchical nature of a set of different microbial communities to determine which members of the bacterial taxonomy are informative so to distinguish between the samples representing different conditions. HFE tackles a number of challenges that accompany the use of microbial compositions as the feature space in classification tasks, including the type of features, which is continuous (relative abundance), the size of the feature space, which is usually thousands of features (OTUs), and the number of categories/classes in the classification task, which can be more than two. If taxonomy depth (seven ranks) is considered a constant, HFE has a time complexity of *O*(*n*(*m* + *m'*)) (see Fig. 2), which makes it suitable for large feature spaces unlike other feature selection methods, such as CFS and wrapper methods, which are computationally expensive and therefore do not scale well. Even in methods based on all-against-all correlation analysis alone the complexity is *O*(*nm*^2^) and thus increasingly infeasible with thousands of features and samples. As illustrated in the Results section, the microbial biomarkers identified by HFE for CRC detection are supported by the evidence previously presented in Zeller et al. and Zackular et al. [2, 3]. Moreover, our results of applying HFE on the CRC dataset provided by Kostic et al. [14] are consistent with their findings of 16S rRNA sequencing analysis, especially with regard to *Fusobacterium* and its associated taxonomic units being enriched in CRC samples compared to normal ones. Our results, as highlighted in Additional file 1: Figure S8, show that high abundance of *Fusobacteriia*, which is at class level, is a potential indicator of CRC. It is worth noting that in Zeller et al. and Zackular et al. [2, 3] the authors report biomarkers at specific taxonomic levels without considering alternatives. In contrast, our findings, as shown in Additional file 1: Figure S8 through Additional file 1: Figure S11, include biomarkers at different taxonomic levels, ranging from phylum to species level in addition to OTUs.

A noteworthy caveat is that the outcome of HFE does not reflect the direction of causality in the respective tasks, but rather sheds light on a number of potential biomarkers that help to distinguish between two or more community types. Similar to mono- vs. polygenic traits in an organism, the identified biomarkers indicate that conditions, such as CRC, adenoma and normal, are often of polymicrobial nature rather than caused by a single microbe and it can vary from a population to another. Classification error rates rise substantially, when a third category—adenoma samples—is included. The confusion, adenoma samples introduce (as shown by the confusion matrices in Additional file 1: Figures S7), indicates that adenoma are associated with a microbiota succession that makes it harder to discern the three categories. Note though that HFE again outperforms the baseline.

HFE is generally applicable to Machine Learning tasks with hierarchically structured feature spaces. Moreover, integrating additional metadata as features in our HFE algorithm is straightforward: after HFE terminates, the feature space can be normally extended with further unstructured features. For Microbiome classification, it seems promising to include functional features with hierarchical nature, e.g. metabolic pathways and enzymes. As for other domains of application, we intend to apply HFE to classification of gene expression datasets using suitable hierarchies of genes, such as Gene Ontology (GO), EC or CAZyme.

## Conclusions

In this study, we tackle the problem of microbiota classification by introducing a hierarchy-based feature engineering method that can handle feature spaces with high dimensionality as in microbiome datasets and exploits the underlying hierarchical structure to construct a much smaller set of informative features for supervised machine learning. The existing hierarchical feature selection algorithms only deal with binary features, which fail to adequately represent biological data and cause a high loss of information compared to relative abundances. Moreover, partial 16S rRNA sequences are often assigned taxonomic ranks to genus or family level with sufficient certainty, which makes their hierarchical information often incomplete. Our Hierarchical Feature Engineering (HFE) method goes beyond mere feature selection into feature engineering as it creates initially an extended version of the feature space, which will be processed through filtering steps to produce a much smaller set of informative features. HFE method can handle continuous features and incomplete hierarchical information. We demonstrate substantial improvements over the baselines (without applying feature selection) and the state-of-the-art microbiota classification tools in terms of classification accuracy, regardless of the actual Machine Learning technique while using drastically reduced feature spaces for Colorectal Cancer Detection. Furthermore, by looking at the variability results among the CRC datasets, we notice that the larger the feature space the smaller the variance of the feature subset size and the larger the feature subset intersection obtained across the different folds in cross validation. This indicates that with large enough training data the HFE’s choices have the potential to be generalized for that classification task. The findings show that HFE makes similar feature choices when applied to different CRC datasets, separately, supporting the attempt to constitute a generalized feature set for CRC detection. Moreover, generalized features bear great explanatory value: they provide a concise description of conditions and thus help to provide pathophysiological insights. Indeed, the automatically and reproducibly derived features are consistent with previously published domain expert analyses.

## Additional file

Additional file 1: **Figure S1.** The PCoA plot of The Human Microbiome Project Consortium (2012) dataset, which is generated via the beta diversity through plots:py script available by QIIME **Figure S2.** The PCoA plot provided in the Meta-analysis of environmental microbiomes conducted by Henschel et al. (2015) **Figure S3.** The PCoA plot of the combined CRC dataset **Figure S4.** Comparison between the baseline and HFE confusion matrices when applied on CRC1 dataset (Zeller et al., 2014) for Cancer vs. Normal classification **Figure S5.** Comparison between the baseline and HFE confusion matrices when applied on CRC2 dataset (Zackular et al., 2014) for Cancer vs. Normal classification **Figure S6.** Comparison between the baseline and HFE confusion matrices when applied on CRC1 + 2 dataset for Cancer vs. Normal classification **Figure S7.** Comparison between the baseline and HFE confusion matrices when applied on CRC1 + 2 **Figure S8.** The taxonomic tree of all the informative features extracted by the HFE method for Cancer vs. Normal classification with respect to the dataset provided by Kostic et al. (2012).dataset for Cancer vs. Normal vs. Adenoma classification **Figure S9.** The taxonomic tree of all the informative features extracted by the HFE method for Cancer vs. Normal classification with respect to CRC1 dataset (Zeller et al., 2014) **Figure S10.** The taxonomic tree of all the informative features extracted by the HFE method for Cancer vs. Normal classification with respect to CRC2 dataset (Zackular et al., 2014) **Figure S11.** The taxonomic tree of all the informative features extracted by the HFE method for Cancer vs. Normal classification with respect to CRC1 + 2 dataset **Table S1.** The cross-validation results of the proposed pipeline when applied for human body site prediction and environment prediction, in terms of AUC. (PDF 27886 kb)

## References

[1] Caporaso JG, Lauber CL, Walters WA, Berg-Lyons D, Lozupone CA (2011). Global patterns of 16s rrna diversity at a depth of millions of sequences per sample. Proc Natl Acad Sci.

[2] Zeller G, Tap J, Voigt AY, Sunagawa S, Kultima JR, Costea PI, Amiot A, Bohm J, Brunetti F, Habermann N, Hercog R, Koch M, Luciani A (2014). Potential of fecal microbiota for early-stage detection of colorectal cancer. Mol Syst Biol.

[3] Zackular JP, Rogers MAM, Ruffin MT, Schloss PD (2014). The human gut microbiome as a screening tool for colorectal cancer. Cancer Prev Res.

[4] Arumugam M, Raes J, Pelletier E, Paslier DL, Yamada T (2011). Enterotypes of the human gut microbiome. Nature.

[5] Costello EK, Lauber CL, Hamady M, Fierer N, Gordon JI, Knight R (2009). Bacterial community variation in human body habitats across space and time. Science.

[6] Henschel A, Anwar MZ, Manohar V (2015). Comprehensive meta-analysis of ontology annotated 16s rrna profiles identifies beta diversity clusters of environmental bacterial communities. PLoS Comput Biol.

[7] Papa E, Docktor M, Smillie C, Weber S, Preheim SP, Gevers D, Giannoukos G, Ciulla D, Tabbaa D, Ingram J, Schauer DB, Ward DV, Korzenik JR, Xavier RJ, Bousvaros A, Alm EJ (2012). Non-invasive mapping of the gastrointestinal microbiota identifies children with inflammatory bowel disease. PLoS One.

[8] Werner JJ, Knights D, Garcia ML, Scalfone NB, Smith S, Yarasheski K, Cummings TA, Beers AR, Knight R, Angenent LT (2011). Bacterial community structures are unique and resilient in full-scale bioenergy systems. PNAS.

[9] Knights D, Costello EK, Knight R (2011). Supervised classification of human microbiota. FEMS Microbiol Rev.

[10] Beck D, Foster JA (2014). Machine learning techniques accurately classify microbial communities by bacterial vaginosis characteristics. PLoS One.

[11] Ditzler G, Morrison JC, Lan Y, Rosen GL (2015). Fizzy: feature subset selection for metagenomics. BMC Bioinformatics.

[12] Pasolli E, Truong DT, Malik F, Waldron L, Segata N (2016). Machine learning meta-analysis of large metagenomic datasets: tools and biological insights. PLoS Comput Biol.

[13] Hall MA (2000). Correlation-based feature selection for discrete and numeric class machine learning. Proceedings of the seventeenth international conference on machine learning. ICML ‘00.

[14] Kostic AD, Gevers D, Pedamallu CS, Michaud M, Duke F, Earl AM, Ojesina AI, Jung J, Bass AJ, Tabernero J (2012). Genomic analysis identifies association of fusobacterium with colorectal carcinoma. Genome Res.

[15] Ristoski P, Paulheim H (2014). Feature selection in hierarchical feature spaces. Discovery. Science.

[16] Caporaso JG, Kuczynski J, Stombaugh J, Bittinger K, Bushman FD, Costello EK, Fierer N, Pena AG, Goodrich JK, Gordon JI, Huttley GA, Kelley ST, Knights D (2010). QIIME allows analysis of high-throughput community sequencing data. Nat Methods.

[17] Friedman J, Alm EJ. Inferring correlation networks from genomic survey data. PLOS Computational Biology. 2012;8(9) 10.1371/journal.pcbi.1002687 PMC3447976 23028285

[18] Mitchell TM (1997). Machine learning, 1st edn.

[19] Fayyad U, Irani K (1993). Multi-interval discretization of continuousvalued attributes for classification learning. IJCAI–93.

[20] Hall M, Frank E, Holmes G, Pfahringer B, Reutemann P, Witten IH (2009). The weka data mining software: an update. ACM SIGKDD Explorations Newsletter.

[21] Bishop C (1995). Neural networks for pattern recognition.

[22] The Human Microbiome Project Consortium (2012). Structure, function and diversity of the healthy human microbiome. Nature.

[23] Feng, Q., Liang, S., Jia, H., Stadlmayr, A., Tang, L., Lan, Z., Zhang, D., Xia, H., Xu, X., Jie, Z., et al.: Gut microbiome development along the colorectal adenoma–carcinoma sequence. Nat Commun 6, 1–13 (2015). 10.1038/ncomms7528 25758642

[24] Dao MC, Everard A, Aron-Wisnewsky J, Sokolovska N, Prifti E (2016). Other: Akkermansia muciniphila and improved metabolic health during a dietary intervention in obesity: relationship with gut microbiome richness and ecology. Gut.

[25] Derrien M, Belzer C, de Vos WM. Akkermansia muciniphila and its role in regulating host functions. Microb Pathog. 2016; 10.1016/j.micpath.2016.02.005. 10.1016/j.micpath.2016.02.005 26875998

