# Supplementary material for: Taxonomy-aware feature engineering for microbiome classification
Source: BMC Bioinformatics. 2018 Jun 15;19:227. doi: 10.1186/s12859-018-2205-3 (PMC6003080; doi:10.1186/s12859-018-2205-3)
Supplement: Supplementary file 1 — Figure S1. The PCoA plot of The Human Microbiome Project Consortium (2012) dataset, which is generated via the beta diversity through plots:py script available by QIIME Figure S2. The PCoA plot provided in the Meta-analysis of environmental microbiomes conducted by Henschel et al. (2015) Figure S3. The PCoA plot of the combined CRC dataset Figure S4. Comparison between the baseline and HFE confusion matrices when applied on CRC1 dataset (Zeller et al., 2014) for Cancer vs. Normal classification Figure S5. Comparison between the baseline and HFE confusion matrices when applied on CRC2 dataset (Zackular et al., 2014) for Cancer vs. Normal classification Figure S6. Comparison between the baseline and HFE confusion matrices when applied on CRC1 + 2 dataset for Cancer vs. Normal classification Figure S7. Comparison between the baseline and HFE confusion matrices when applied on CRC1 + 2 Figure S8. The taxonomic tree of all the informative features extracted by the HFE method for Cancer vs. Normal classification with respect to the dataset provided by Kostic et al. (2012).dataset for Cancer vs. Normal vs. Adenoma classification Figure S9. The taxonomic tree of all the informative features extracted by the HFE method for Cancer vs. Normal classification with respect to CRC1 dataset (Zeller et al., 2014) Figure S10. The taxonomic tree of all the informative features extracted by the HFE method for Cancer vs. Normal classification with respect to CRC2 dataset (Zackular et al., 2014) Figure S11. The taxonomic tree of all the informative features extracted by the HFE method for Cancer vs. Normal classification with respect to CRC1 + 2 dataset Table S1. The cross-validation results of the proposed pipeline when applied for human body site prediction and environment prediction, in terms of AUC. (PDF 27886 kb) [file 12859_2018_2205_MOESM1_ESM.pdf]

Additional file 1

1 Additional Figures

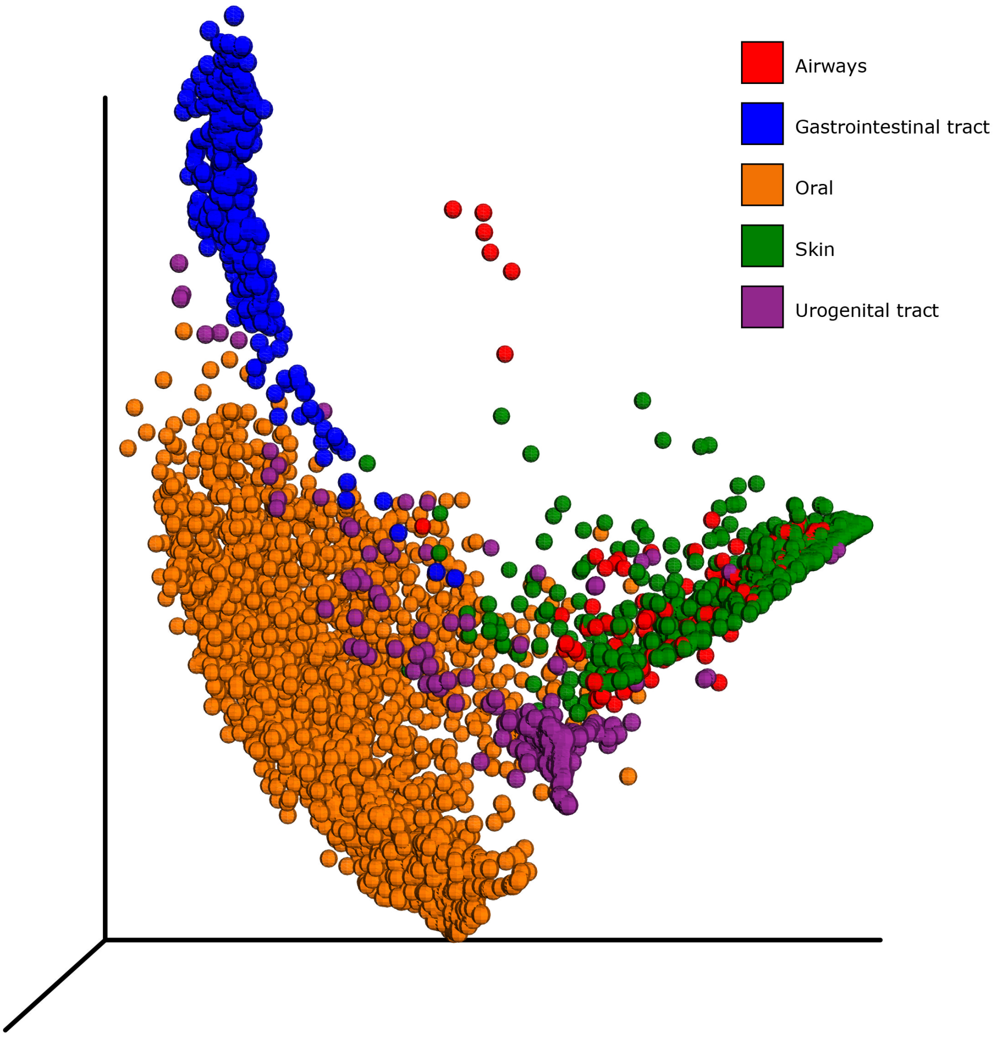

Figure **S1**: The PCoA plot of The Human Microbiome Project Consortium (2012) dataset, which is generated via the *beta_diversity_through_plots.py* script available by QIIME.

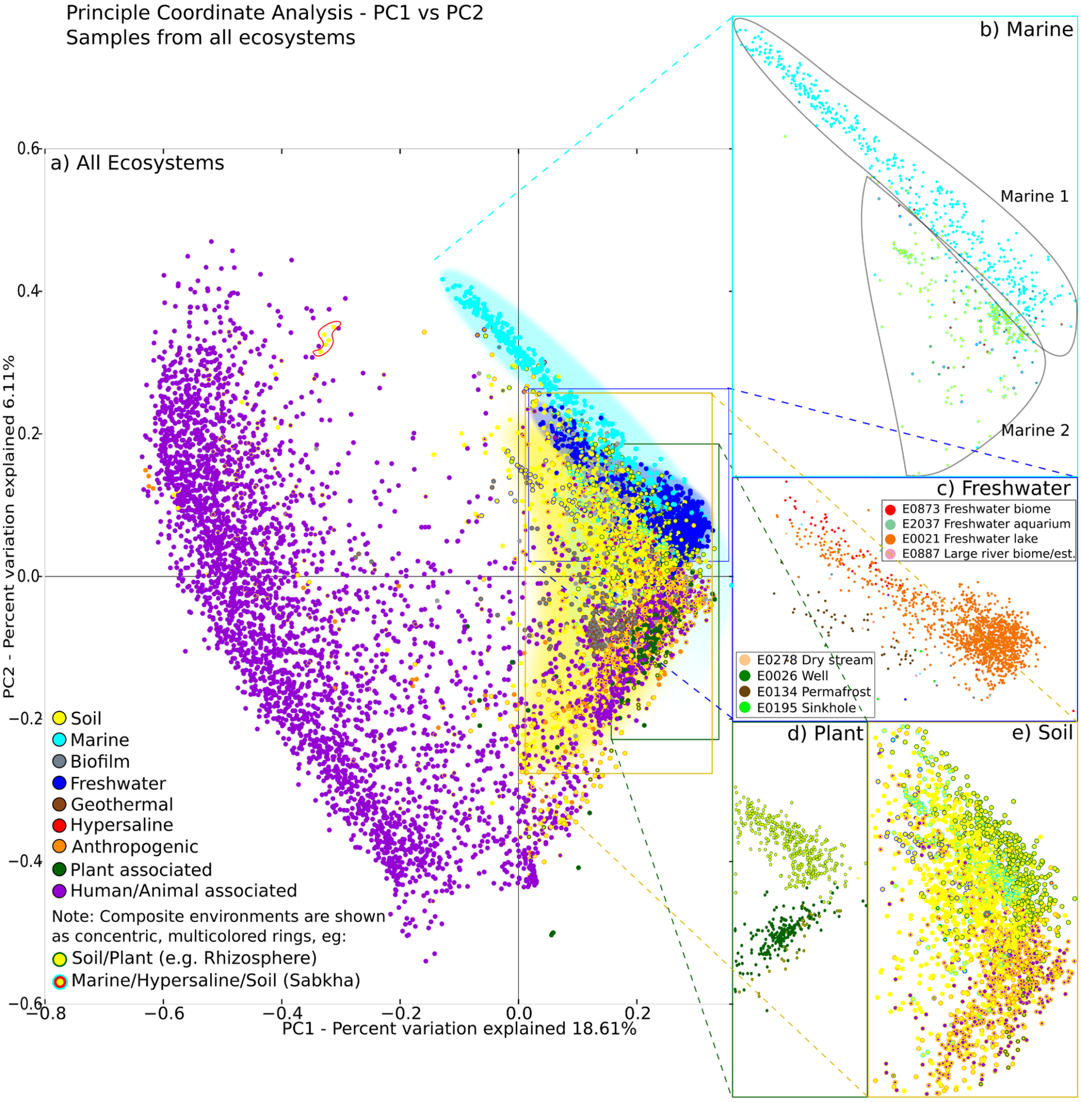

Figure **S2**: The PCoA plot provided in the Meta-analysis of environmental microbiomes conducted by Henschel *et al.* (2015).

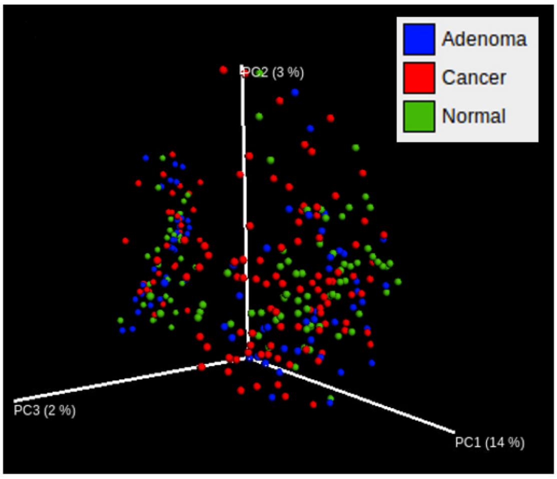

Figure **S3**: The PCoA plot of the combined CRC dataset.

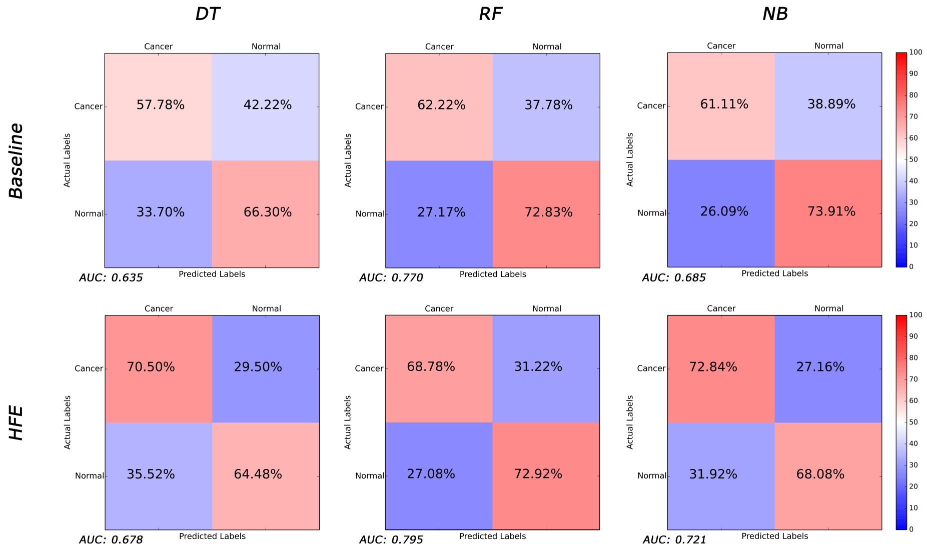

Figure **S4**: Comparison between the baseline and HFE confusion matrices when applied on CRC1 dataset (Zeller *et al.*, 2014) for Cancer vs. Normal classification.

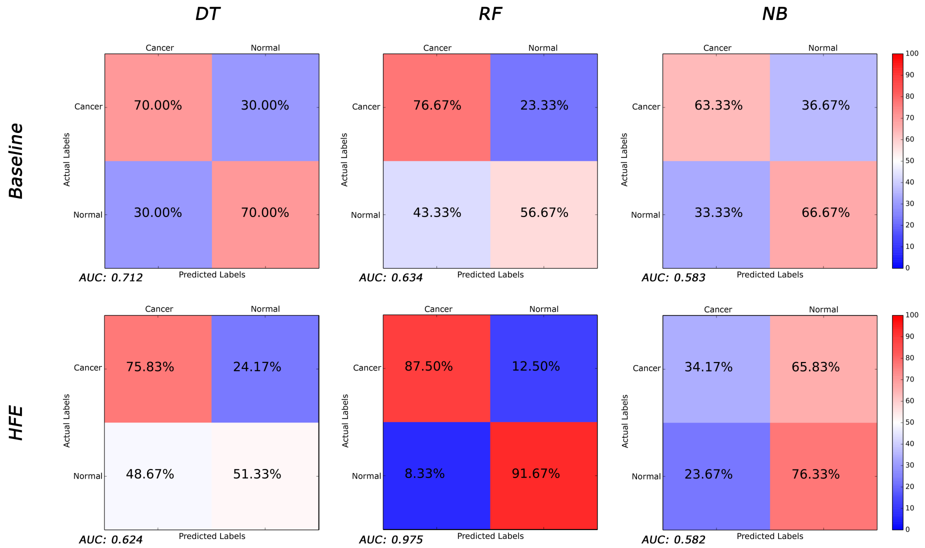

Figure **S5**: Comparison between the baseline and HFE confusion matrices when applied on CRC2 dataset (Zackular *et al.*, 2014) for Cancer vs. Normal classification.

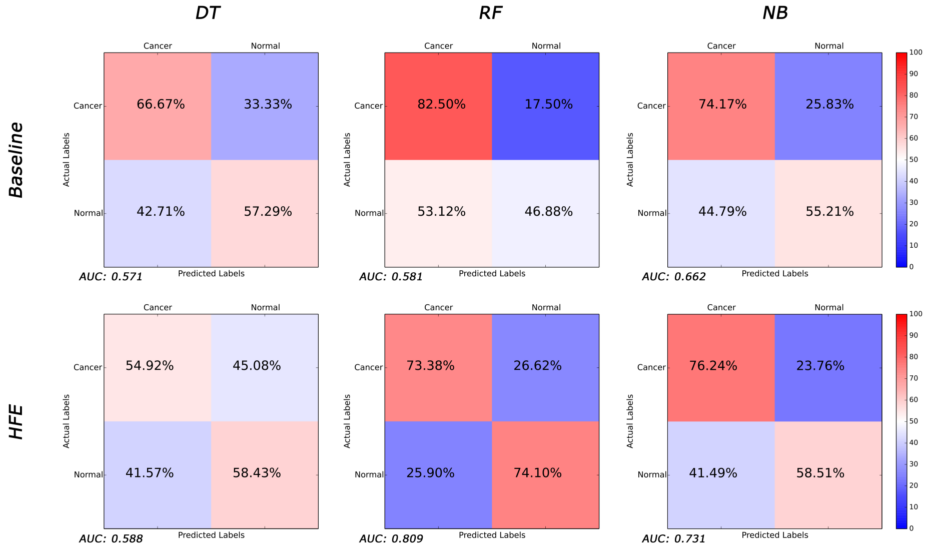

Figure **S6**: Comparison between the baseline and HFE confusion matrices when applied on CRC1+2 dataset for Cancer vs. Normal classification.

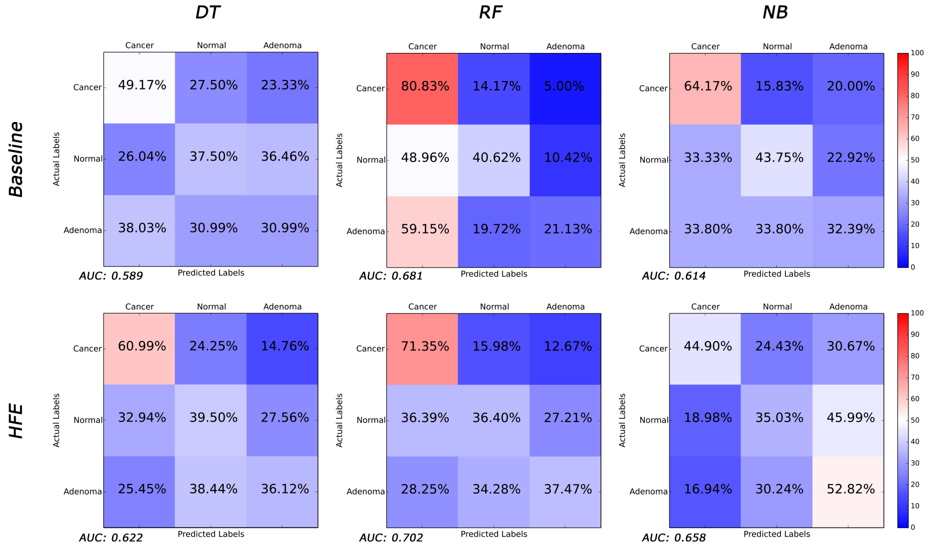

Figure **S7**: Comparison between the baseline and HFE confusion matrices when applied on CRC1+2 dataset for Cancer vs. Normal vs. Adenoma classification.

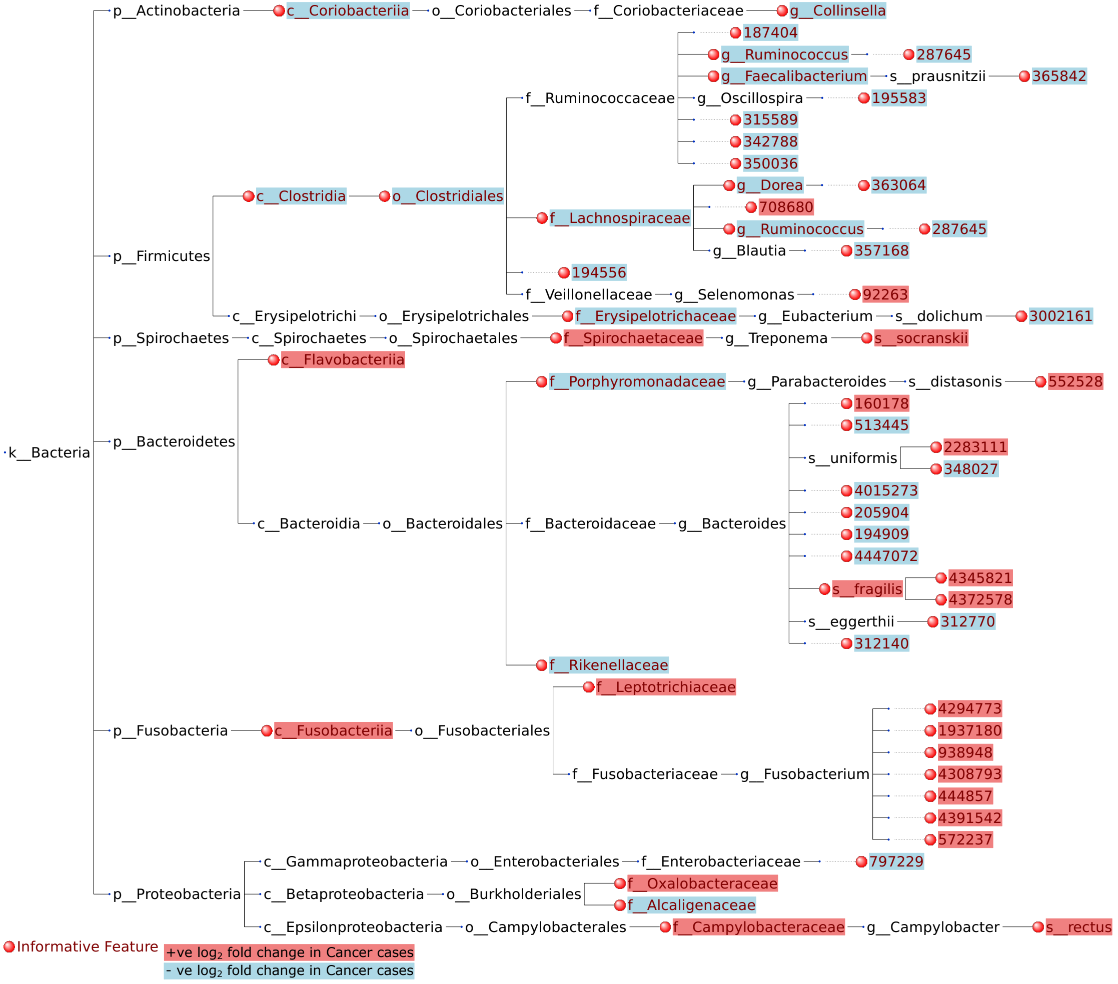

Figure **S8**: The taxonomic tree of all the informative features extracted by the HFE method for Cancer vs. Normal classification with respect to the dataset provided by Kostic *et al.* (2012).

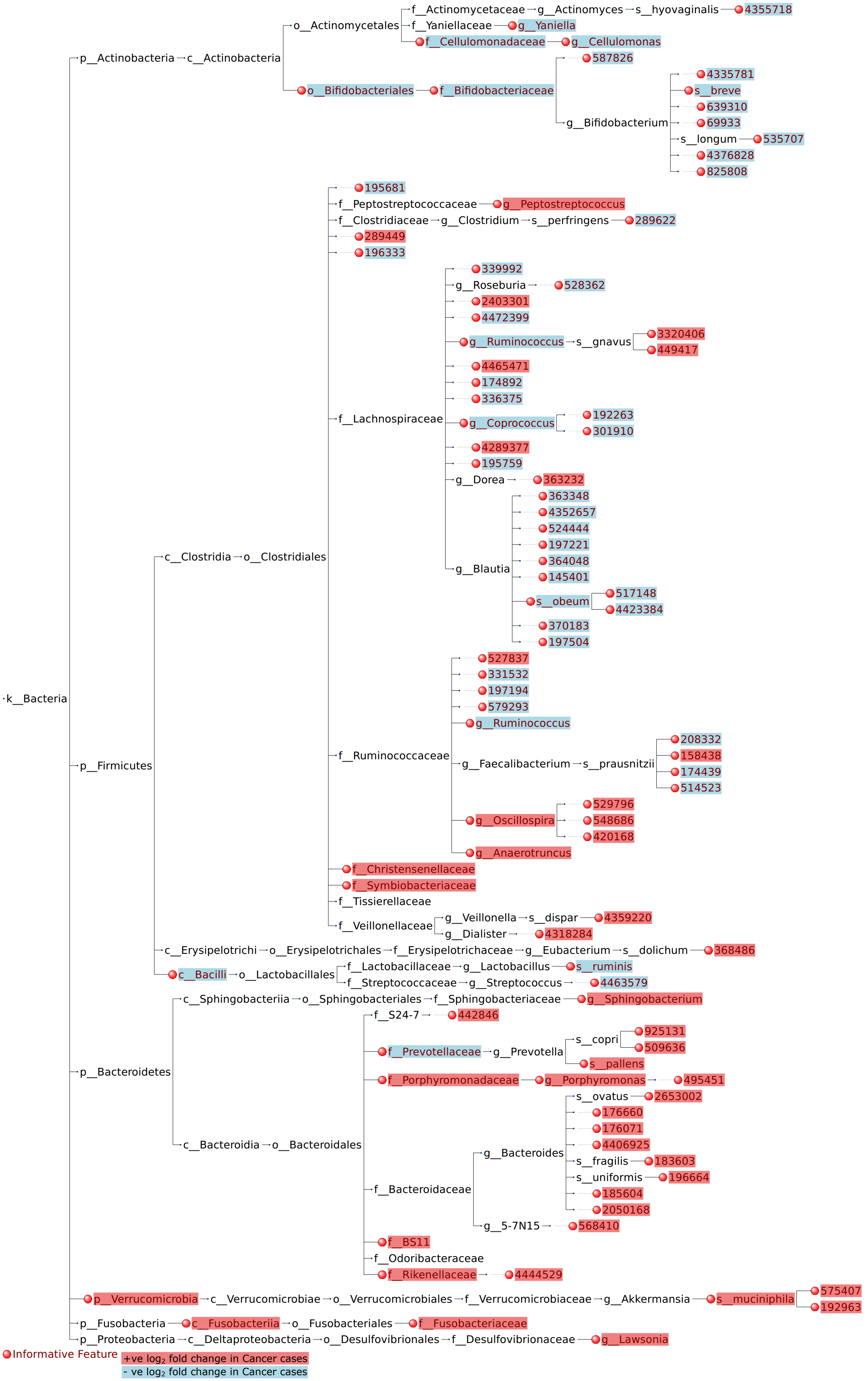

Figure **S9**: The taxonomic tree of all the informative features extracted by the HFE method for Cancer vs. Normal classification with respect to CRC1 dataset (Zeller *et al.*, 2014).

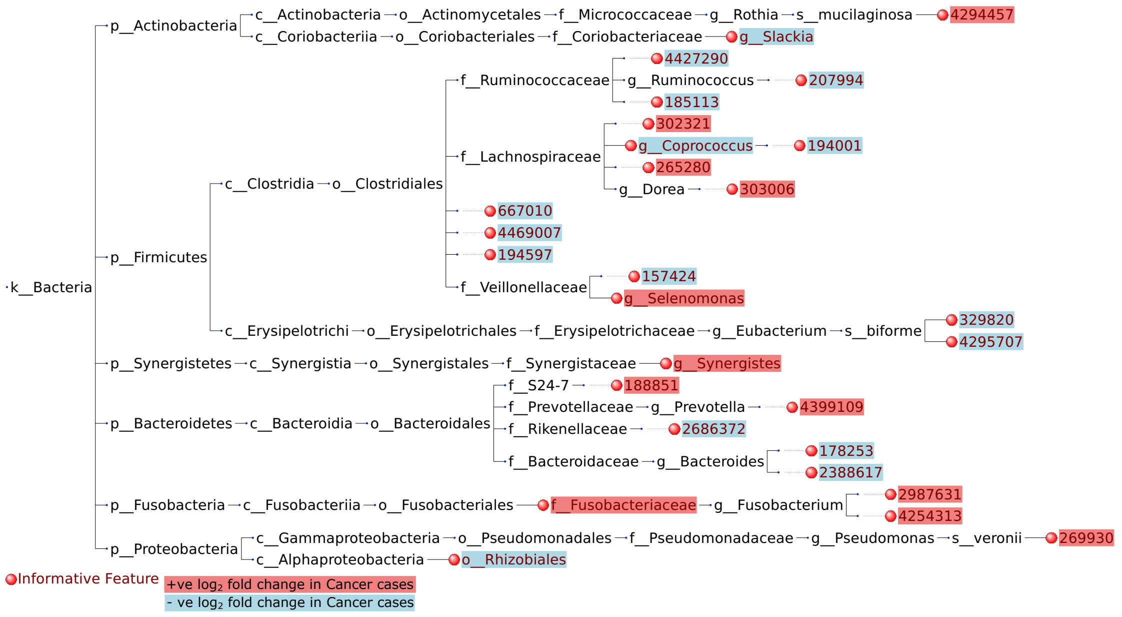

Figure **S10**: The taxonomic tree of all the informative features extracted by the HFE method for Cancer vs. Normal classification with respect to CRC2 dataset (Zackular *et al.*, 2014).

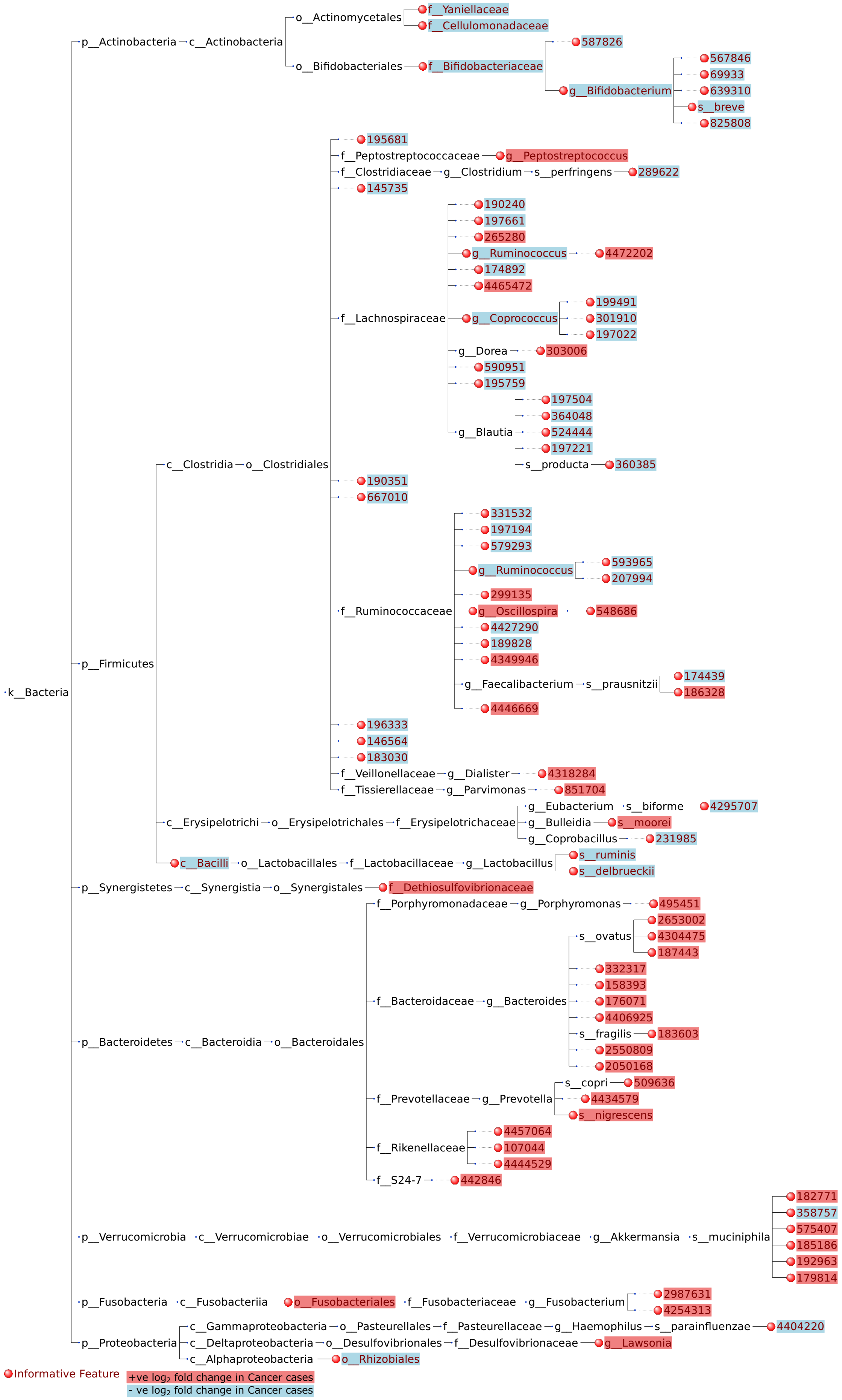

Figure **S11**: The taxonomic tree of all the informative features extracted by the HFE method for Cancer vs. Normal classification with respect to CRC1+2 dataset.

2 Supplementary Tables

Table **S1**: The cross-validation results of the proposed pipeline when applied for human body site prediction and environment prediction, in terms of AUC.

	**Human Body Site Prediction**		**Environment Prediction**	
**ML**	BL	HFE	BL	HFE
**DT**	0.992	0.985	0.973	0.960
**RF**	0.999	0.999	0.999	0.999
**NB**	0.994	0.995	0.903	0.949
**#Features**	5,430	84	30,860	267

BL and HFE refer to the baseline and HFE feature sets, respectively.

References

Henschel, A., Anwar, M., and Manohar, V. (2015). Comprehensive meta-analysis of ontology annotated 16s rrna profiles identifies beta diversity clusters of environmental bacterial communities. *PLoS Computational Biology*, **11**, 1–24.

Kostic, A. D., Gevers, D., Pedamallu, C. S., Michaud, M., Duke, F., Earl, A. M., Ojesina, A. I., Jung, J., Bass, A. J., Tabernero, J., *et al.* (2012). Genomic analysis identifies association of fusobacterium with colorectal carcinoma. *Genome research*, **22**(2), 292–298.

The Human Microbiome Project Consortium (2012). Structure, function and diversity of the healthy human microbiome. *Nature*, **486**, 207–214.

Zackular, J., Rogers, M., Ruffin, M., and Schloss, P. (2014). The human gut microbiome as a screening tool for colorectal cancer. *Cancer Prevention Research*, **7**, 1112–1121.

Zeller, G., Tap, J., Voigt, A., Sunagawa, S., Kultima, J., Costea, P., Amiot, A., Bohm, J., Brunetti, F., Habermann, N., Hercog, R., Koch, M., Luciani, A., *et al.* (2014). Potential of fecal microbiota for early-stage detection of colorectal cancer. *Molecular Systems Biology*, **10**, 1–18.
